# Self-Healing Polymeric Materials and Composites for Additive Manufacturing

**DOI:** 10.3390/polym15214206

**Published:** 2023-10-24

**Authors:** Yixue Jiang, Evelyn Ling Ling Ng, Danielle Xinyun Han, Yinjia Yan, Siew Yin Chan, John Wang, Benjamin Qi Yu Chan

**Affiliations:** 1 Institute of Materials Research and Engineering (IMRE), Agency for Science, Technology and Research (A*STAR), 2 Fusionopolis Way, Innovis #08-03, Singapore 138634, Singapore; 2 Department of Materials Science and Engineering, College of Design and Engineering, National University of Singapore, 9 Engineering Drive 1, Singapore 117575, Singapore; 3 Frontiers Science Center for Flexible Electronics (FSCFE), Xi’an Institute of Flexible Electronics (IFE), Xi’an Institute of Biomedical Materials and Engineering (IBME), and Ningbo Institute, Northwestern Polytechnical University, 127 West Youyi Road, Xi’an 710072, China

**Keywords:** 3D printing, self-healing, polymers and composites, additive manufacturing, smart polymers

## Abstract

Self-healing polymers have received widespread attention due to their ability to repair damage autonomously and increase material stability, reliability, and economy. However, the processability of self-healing materials has yet to be studied, limiting the application of rich self-healing mechanisms. Additive manufacturing effectively improves the shortcomings of conventional processing while increasing production speed, accuracy, and complexity, offering great promise for self-healing polymer applications. This article summarizes the current self-healing mechanisms of self-healing polymers and their corresponding additive manufacturing methods, and provides an outlook on future developments in the field.

## 1. Introduction

Inspired by nature, researchers have introduced self-healing mechanisms into materials, enabling them to repair internal or external damage, regain functionality, and extend their lifetime. These are called self-healing materials [1,2,3,4,5]. Self-healing materials are regarded as a new generation of advanced materials, and have been applied in many fields due to their advantages [6,7,8]. It has attracted the attention of modern science, materials engineering, and commodity research over the last decades [7,9,10]. Self-healing polymers endow fabricated products with product durability, mechanical stability, and reliability, as well as having a low environmental impact [6,9]. For example, soft robots, which are susceptible to damage, commonly require damaged components to be replaced entirely. This is costly, time consuming, and waste generating. Developing soft robots using self-healing polymers as an alternative presents a more economical and sustainable solution [11,12,13].

Generally, self-healing mechanisms of self-healing materials fall into two categories: extrinsic healing and intrinsic healing. Extrinsic healing includes microcapsules, microvascular networks, and nanoparticles. In contrast, intrinsic healing is divided into dynamic covalent bonding types, including Diels–Alder reaction, disulfide, and dynamic non-covalent bonding, involving hydrogen bonds, ionic interactions, and coordination.

Although the development of self-healing materials has attracted increasing interest, reports have focused on studies of healing ability and underlying mechanisms, with very little attention paid to their processability [14,15]. In addition, traditional processing techniques such as casting, electrospinning, or extrusion limit the complexity, heterogeneous material integration, precision, and yield of objects, while manufacturing with three-dimensional printing (3D printing) can effectively improve these deficiencies [12,16,17,18,19]. Three-dimensional printing, also referred to as additive manufacturing (AM), fabricates 3D physical objects consistent with corresponding digital models by stacking materials layer-by-layer, without the need for molds or machining [15,20,21].

Three-dimensional printing has many advantages, such as fast production speeds and the ability to precisely control structures’ dimensions, shape, and density, allowing for the mass customization of complex equipment [12,22]. At the same time, because no additional tools or secondary processing is required, the effect of reducing manufacturing waste, and saving production time and energy consumption can be achieved [22,23]. However, the evaluation of self-healing polymers in the context of additive manufacturing (AM) necessitates a thorough assessment of various performance metrics. Of greatest importance is the healing efficiency, which serves as a measure of the material’s ability to restore its properties after sustaining damage. In order to ensure structural integrity, it becomes imperative to maintain mechanical properties such as strength and stiffness. However, striking a delicate balance between maintaining a reasonable viscosity—an essential requirement for resin-based 3D printing—and preserving optimal healing efficiency proves to be quite challenging.

Manufacturing self-healing materials with 3D printing can combine the advantages of both, facilitating the manufacture of smart devices and enabling a more comprehensive range of applications for self-healing materials. These applications include, for example, aerospace applications where they hold promise in reducing maintenance requirements for critical components; biomedical applications where they enhance device lifespan leading to improved patient outcomes; automotive manufacturing, which stands to gain from impact-resistant parts; consumer electronics benefiting from durable self-healing coatings; and infrastructure sector enjoying reduced maintenance costs along with enhanced durability. The advent of additive manufacturing has revolutionized these domains by enabling customization options, facilitating production complexities inherent in intricate geometries while ensuring on-demand fabrication capabilities resulting in heightened operational efficiency and resilience [16,24,25].

So far, the 3D printing methods reported for the manufacturing of self-healing materials have been divided into extrusion-based AM processes, including mainly fused deposition modelling (FDM) and direct-ink writing (DIW), and vat photopolymerized AM processes, primarily including stereolithography (SLA) and digital light processing (DLP). With research in this field becoming more extensive and comprehensive, there is a need now to review and summarize existing AM methods of self-healing materials, as well as its projected applications, to ensure its long-term relevance. This review focuses on various self-healing mechanisms and 3D printing techniques, as well as an analysis of the compatibility between them. Readers are directed to other detailed reviews on self-healing materials in related fields such as sensors [26], soft robotics [11], material design [27,28], etc. The review is organized into three parts: firstly, on main self-healing mechanisms; secondly, an overview of the leading 3D printing methods used at this stage for self-healing polymers; thirdly, proposed applications for future additive manufacturing of self-healing polymers. A brief summary of existing work on 3D-printable self-healing materials classified into their various printing and self-healing mechanisms are consolidated in Table 1.

## 2. Self-Healing Mechanisms

A wide range of self-healing mechanisms have been developed since the 1990s when Dry et al. researched the development of self-healing fibre-reinforced composites and polymeric smart materials [45]. In 2001, White et al. investigated the microencapsulation of healing agents embedded in polymeric matrices [10,45,46]. Self-healing mechanisms can be classified as extrinsic or intrinsic. The healing ability of extrinsic self-healing materials arise from integrating self-healing properties into the original material system. In contrast, intrinsic self-healing materials depend on the material’s inherent chemical groups and properties [11].

### 2.1. Extrinsic Healing

#### 2.1.1. Capsule-Based Healing Systems

**Figure 1:**
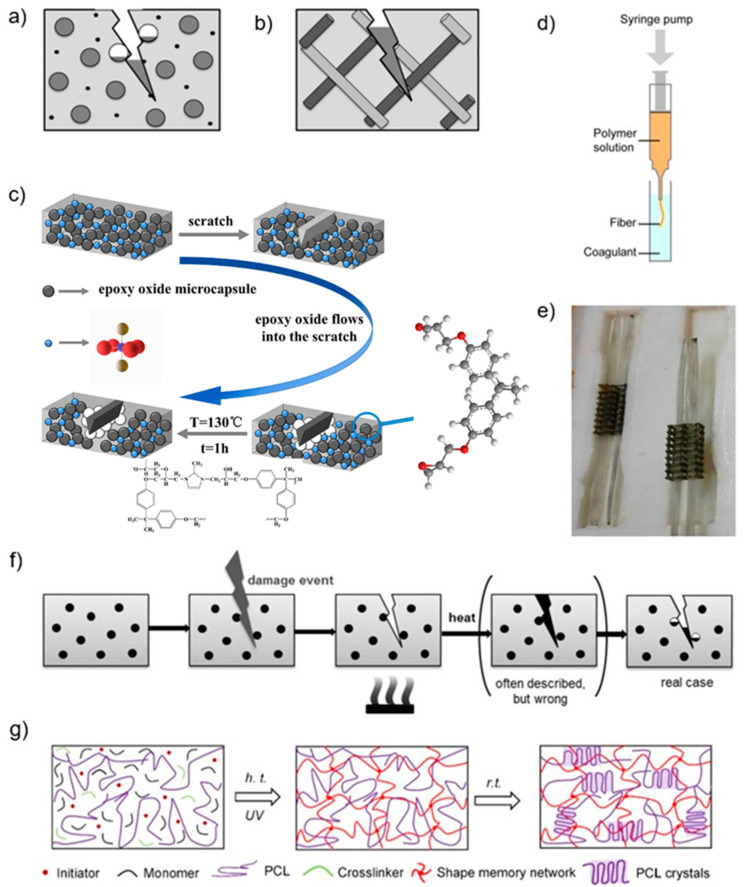
(**a**) Capsule-based healing systems, the gray ball is the healing agent, and the black ball is the catalyst or curing agent [9]. (**b**) Vascular-based healing systems, the gray ball is the healing agent, and the dark gray ball is the catalyst or curing agent [9]. (**c**) Schematic diagram of scratch repair principle with MF microcapsules embedded in epoxy oxides [47]. (**d**) Schematic diagram of 3D-printed vascularized fibres [52]. (**e**) Microvascular networks infused with self-healing and curing agents are embedded in silicon moulds [50]. (**f**) Phase-separated additives healing systems, thermoplastic particles (black balls) are dispersed in the matrix [9]. (**g**) Chemical structure evolution of the repair principle of the SH-SMP solution during UV-based 3D printing at high temperature (h.t.) and cooling down to room temperature (r.t.) [40].

The most common extrinsic self-healing methods utilize micro containers made from brittle polymers, such as microcapsules containing a healing agent, appropriate catalyst, curing agent, and reaction initiator embedded into the polymer matrix [9,10]. During object fracture, the micro containers are broken, automatically releasing healing agents into the crack site and filling the damaged area (Figure 1a) [10,37]. This method is often used to enable materials with stiff polymer matrices to achieve self-healing. The damaging force (trigger) is required to activate the healing action rather than the molecular diffusion of the matrix [11,37]. One advantage of microencapsulation technology is that the many different healing chemistries and encapsulation techniques allow the healing mechanism to be adapted to different matrices. However, the volume of healing agent is limited, limiting healing to microscopic damage, and/or it can only occur once [9,11].

Recently, Ma et al. embedded melamine–formaldehyde (MF) microcapsules, which wrap the epoxy oxide as a repairing agent, and Cu(MI)_4_Br_2_ as a curing agent in an epoxy oxide-based self-healing system, and printed out samples using a 3D printer (Figure 1c) [47]. The curing agent decomposed above the decomposition temperature and cured the damaged area for repair. It was found that adding 10 wt% microcapsules to the matrix could improve the tensile strength of the 3D-printed samples. After heating the scratches, the mechanical strength of the fractured material could be restored to 44.42 MPa with a high self-healing efficiency of 89.98% [47]. The 4D printing microencapsulated epoxy oxide self-healing system in the study was shown to form self-healing materials with high tensile strength and stability properties. This is beneficial to various practical 4D printing-related applications and shows excellent potential biological, medical, and bionic applications [47].

#### 2.1.2. Vascular-Based Healing Systems

Microvascular healing systems store healing agents in hollow channels or interconnected networks and heal on a similar principle to microcapsules, i.e., that injury triggers self-healing (Figure 1b). The difference is that they can store more healing agent and transport it over greater distances. Hence, more significant injuries may be repaired, and reparation can occur multiple times within a particular area [9].

However, due to the solid nature of the vascular system, it cannot be used directly in resins or inks, and cannot be directly 3D-printed to obtain the self-healing product. More experiments were performed using 3D-printed vascular systems in combination with other methods, such as casting to obtain self-healing objects (Figure 1d,e) [48,49,50].

#### 2.1.3. Phase-Separated Additives Healing Systems

Self-healing can also be achieved by embedding solid thermoplastic particles with a low melting temperature in the matrix. At a temperature above its T_g_, thermoplastic additives melt and diffuse into the interfaces of the crack, and achieve self-healing by adhering the two surfaces together as they recrystallizes [30,51]. Unlike the encapsulation approach, this healing process is non-autonomous because thermal activation is needed, and can perform several times on the same fracture surface [9,11]. The disadvantage of nanoparticles is similar to microcapsules in that the thermoplastic particles do not entirely fill the damaged area, limiting the healing effect [9] (Figure 1f).

Zhang et al. successfully incorporated the semicrystalline linear polymer polycaprolactone (PCL) into a methacrylate-based shape memory polymer (SMP) system. They printed complex structures with self-healing properties with a DLP 3D printer [40]. PCL acts as a self-healing agent to provide self-healing properties through the above principles and can recover the mechanical properties of damaged structures to over 90% [40] (Figure 1g). Similar to Zhang et al.’s experimental approach, Peng et al. printed a highly extensible, self-healing shape memory elastomer by a desktop FFF 3D printer, and the self-healing properties were achieved by semicrystalline thermoplastic PCL, which was incorporated into the thermoplastic elastomer [30].

### 2.2. Intrinsic Self-Healing

More researchers are combining intrinsic self-healing with 3D printing because of its theoretically infinite healing capacity [53]. Self-healing behavior is triggered by damage, driven by surface tension and elastic energy of stress sources or by appropriate external stimuli such as temperature, heat, electromagnetic radiation, pH, light. or ionic strength changes, and can be permanently fractured and reorganized [9,10]. Due to the different types of interactions used to achieve healing, intrinsic healing mechanisms can be further subdivided into those based on covalent interactions, such as the Diels–Alder reaction and disulfide; or non-covalent interactions, such as hydrogen bonding, ionic interactions, and coordination [1,9]. Due to the high bond strength of 150–550 kJ mol^−1^, the breakage and reformation of covalent bonds is usually non-autonomous and requires relatively large amounts of energy from external stimuli. Still, they typically exhibit better elasticity and durability. Non-covalent bonds usually result from intermolecular interactions, and the many non-covalent bonds that crosslink polymers can lead to the formation of supramolecular networks [11]. Compared to dynamic covalent chemistry, non-covalent bond strengths are lower. This weaker crosslink means that less energy is required to break these bonds, being more susceptible to the external environments, thus allowing materials to exhibit extraordinary healing and stimuli-responsive properties [1,43].

#### 2.2.1. Dynamic Covalent Bonds

##### Diels–Alder Reactions

**Figure 2:**
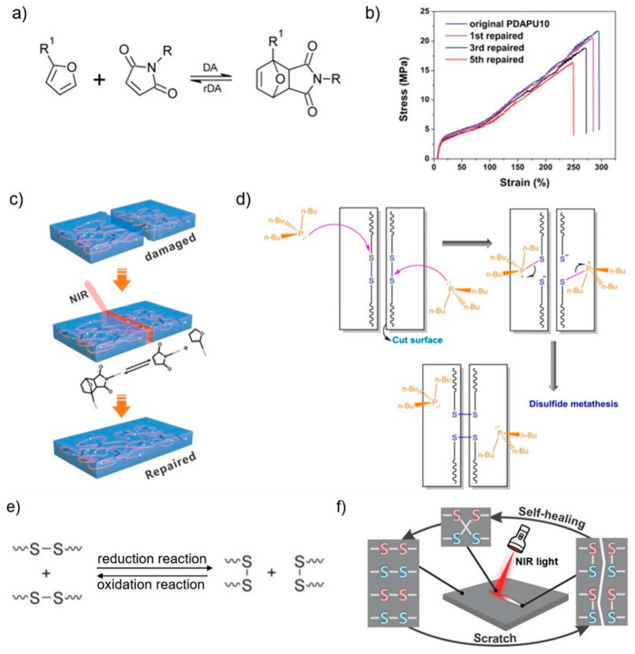
(**a**) Schematic diagram of reversible DA reaction between furan and maleimide groups [9]. (**b**) Stress–strain curve of the self-healing material (named PDAPU10) designed by Yang et al. for five stretch–recovery cycles with a self-healing efficiency of 92% [16]. (**c**) Schematic representation of DA-reaction-based precise self-healing triggered by NIR [16]. (**d**) Schematic of the disulfide metathesis reaction with catalyst TBP [57]. (**e**) Schematic of disulfide interchange reaction [54]. (**f**) Schematic of disulfide-bonds-based self-healing response activated by NIR [42].

The most common dynamic covalent bonds are thermoreversible Diels–Alder (DA) reactions, whose bond strength is relatively high, so its healing process usually occurs at high temperatures [11,54]. DA reactions are usually formed by equilibrium reactions between dienes and dienophiles, both present as functional groups on the constituting monomers (or prepolymers), the most common of which is the reversible DA cycloaddition between furans and maleimides (Figure 2a) [36]. DA reactions require high temperatures to activate healing. On one hand, this shifts the equilibrium of the exothermic DA reaction from the major part of the DA bonds formed at ambient temperature to the breaking of these bonds, leading to higher concentrations of reactive dienes and dienophilic functional groups. On the other hand, this increases molecular mobility, further facilitating contact between the fracture surfaces and facilitating self-healing [11]. For example, the furan/maleimide DA polymer network requires thermal activation at 80–130 °C, including decoupling of the furan/maleimide pair when temperatures above the dissociation temperature (TD), molecular movement in the polymer network, and subsequent furan/maleimide coupling at the condensation temperature [32].

It has been shown that macroscopic damage can heal with very high healing efficiency by this mechanism, and that multiple damage–healing cycles can be performed without a significant decrease in healing performance (Figure 2b) [16]. In addition to self-healing through high-temperature activation of molecular segments, Yang et al. achieved the process of self-healing triggered by near-infrared (NIR) light through photothermal conversion and the method can be remotely controlled to irradiate the self-healing area precisely without damaging their original 3D structures (Figure 2c) [16]. The mechanism can also be introduced into 3D printing, and it has been reported that by introducing the DA response into the new 3DP ink, DIW prints maintain their excellent self-healing properties [16,36].

Diels–Alder reactions, while promising for intrinsic self-healing systems in polymers and materials, have some limitations. These include temperature sensitivity, reversibility issues, slow kinetics at lower temperatures, stoichiometric requirements, limited chemical compatibility, environmental sensitivity, potential brittleness, and limited reusability [55]. These challenges can impact the efficiency, speed, and applicability of Diels–Alder-based self-healing materials in various real-world applications. Ongoing research aims to address these limitations by developing innovative catalysts, more reversible reaction mechanisms, and improved material designs [56].

##### Disulfide Bonds

Among the intrinsic methods, the self-healing effect of disulfide bonds has attracted much attention due to its ability to allow the full recovery of mechanical properties under mild healing conditions [41,54]. The disulfide groups can be cleaved by external forces, reduction reaction, etc., to form two thiol groups and can be reformed under stimulation for disulfide metathesis, which is responsible for the self-healing properties (Figure 2e) [54]. Disulphide metathesis reactions can be accelerated by catalysts. For example, Qureshi et al. managed to successfully reduce the reaction time by adding tributylphosphine (TBP) to a self-healing UV-cured ink [57]. In this experiment, the nucleophilic attack of TBP on the disulfide bond produced a TBP cationic intermediate and a thiolate anion [57,58]. The cross-nucleophilic attack of the thiolate anions on the other sulfur atoms occurs via the intermediate, leading to the exchange of network chains and the return of the catalyst to its original state, where the disulfide metathesis reaction is repeated (Figure 2d) [57,58]. Due to the limited mobility of chains in glassy polymers, the self-healing process needs to take place in the mobile chain segments above the T_g_ for the interchange reaction to take place. Hence, this method is widely used for low T_g_ materials such as polyurethanes and polyesters [54].

Li et al. introduced disulfide bonds into polyurethane acrylates and printed them by digital light processing 3D printing technology. The resulting polyurethane elastomer showed good self-healing ability, with the healed samples recovering to 95% of their original strength after 12 h of healing at 80 °C, and could be healed multiple times [41]. In the same vein, Yu et al. designed a photo elastomer ink containing disulfide groups that can achieve self-healing properties by disulfide metathesis reaction, and elastomers obtained by 3D printing can heal themselves wholly and quickly [59]. Similar to the Diels–Alder reaction, dynamic disulfide bonds can be used to exploit the principle of photothermal conversion through a self-healing process triggered by near-infrared (NIR) light (Figure 2f) [42].

However, it is noteworthy that disulfide-based reactions still exhibit certain limitations. These include relatively slow kinetics, temperature sensitivity, potential catalyst dependency, stoichiometry requirements, sensitivity to environmental factors, limited chemical compatibility, and issues related to durability and reusability. These limitations can affect the speed, efficiency, and applicability of disulfide-based self-healing, especially in environments with temperature fluctuations or when rapid repairs are essential [11]. Nevertheless, ongoing research seeks to overcome these challenges by developing more effective catalysts, optimized reactant designs, and more resilient materials, enhancing the potential of disulfide reactions for self-healing applications [60].

#### 2.2.2. Non-Covalent Interactions

##### Hydrogen Bonds

**Figure 3:**
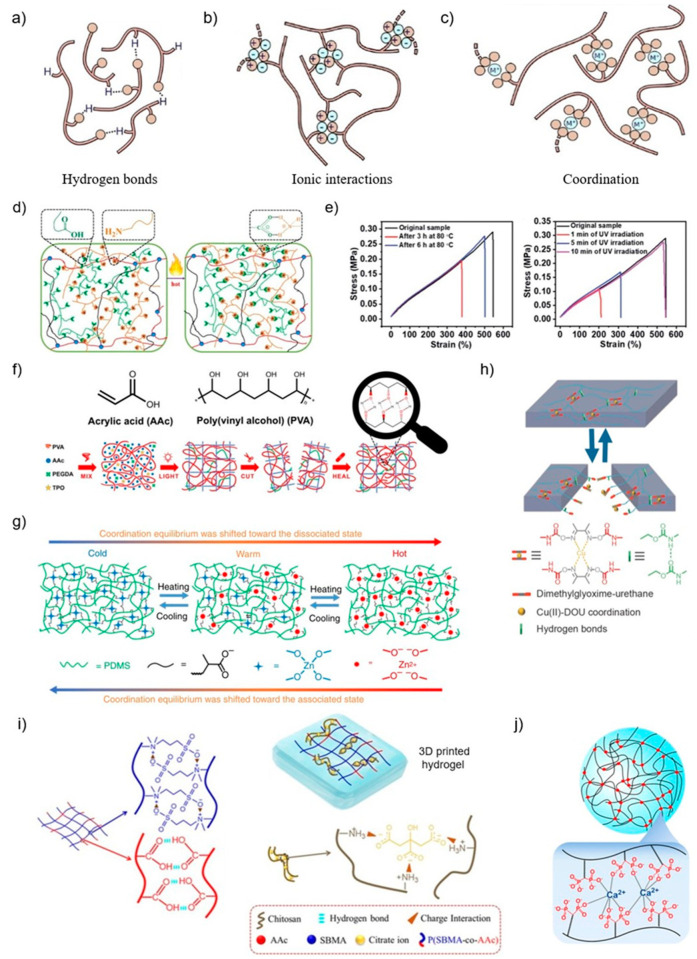
(**a**–**c**) Non-covalent interactions, including hydrogen bonding, ionic interactions, or coordination metal–ligand complexes [11]. (**d**) Schematic illustration of the dynamic ionic crosslinked network between the carboxyl and amino-functional polysiloxanes [39]. (**e**) Stress–strain curves of original and healed samples at 80 °C for 3 and 6 h, with healing efficiency over 95%. Stress–strain curves of original and healed samples at 1, 5, and 10 min of UV irradiation, with healing efficiency over 99% [43]. (**f**) Schematic of the chemical structure of AAc and PVA in a light-curing resin, the multiple hydrogen bonds formed, and the self-healing process [44]. (**g**) Schematic structure of the polymer network for self-healing through Zn(II)–carboxylate coordination bonding [34]. (**h**) Schematic of triple dynamic self-healing of Cu–DOU–CPU polymers [33]. (**i**) Schematic diagram of the self-healing principle of P(SBMA-co-Ac)/CS–Cit DN hydrogels [74]. (**j**) Schematic presentation of a hydrogel network formed by HA−BP macromolecules upon coordination bonding with Ca^2+^ ions [45].

The strength of hydrogen bonds varies from 2 to 40 kcal mol^−1^, depending on the hydrogen donor and acceptor [61,62]. Due to its low bond strength, hydrogen-bonded SH polymers can easily undergo the self-healing process by increasing temperatures, as high temperatures result in the breakage of hydrogen bond crosslinks and lead to viscous flow, thereby contributing to self-healing. This is in addition to the relatively fast reformation of hydrogen bonds due to their affinity, offering the possibility of developing autonomous self-healing polymers (Figure 3a) [7,11,63]. Some substances can be repaired after macroscopic damage, even for non-functionalized substances, due to the presence of their hydrogen bonds, e.g., polyurethanes, where interfacial healing is efficiently achieved mainly through hydrogen bonding among urethane units at the broken interface and the highly efficient healing properties are also retained even after 3D printing [10,14,64].

However, low-bond-energy crosslinks are detrimental to mechanical strength, strain recovery, and creep behavior. To improve mechanical strength and structural stability, multivalent hydrogen bonds can be utilized, increasing the number of hydrogen bonds formed per crosslink, and then increasing the strength of the crosslinks [11,65]. This theory is used in many 3D printing materials to provide self-healing properties. For example, Caprioli et al. mixed unmodified non-crosslinked poly (vinyl alcohol) (PVA) with acrylic acid (AAc), the cross-linker poly (ethylene glycol) diacrylate (PEGDA), and photoinitiator diphenyl (2,4,6-trimethylbenzoyl)phosphine oxide (TPO) to make a photocurable ink [44]. PVA has the inherent property of chain interdiffusion and the presence of hydroxyl groups formed by hydrogen bonds, and the carboxylic groups of AAc can form multiple hydrogen bonds with PVA chains. These give the 3D-printed hydrogel its high self-healing property (Figure 3f) [44,66].

Ureidopyrimidinone (UPy) methacrylate monomers are also often used in 3D-printed polymer matrices because of their ability to form four hydrogen bonds with a self-complementary unit, responsible for the self-healing behavior [13]. Alternatively, a healing agent can be added to the matrix to exploit hydrogen bonding interactions. For example, Triton X-100 can be used as a healing agent because of the hydrogen bonding of its hydrophilic polyethene oxide parts [67]. For example, Kee et al. added Triton X-100 to 3D-printable thermoelectric composites, allowing the fractured material to retain over 85% of its properties after healing [67].

##### Ionic Interactions

Self-healing polymers based on supramolecular dynamic networks can also be made using ionic interactions between ionic polymers [11]. Typically, ionomers are polymer chains partially modified with ionic side groups and the corresponding counter ions (Figure 3b) [9]. The ionomers contain between 1 and 15% of charged or ionic species. These ionic groups tend to aggregate, forming ionic clusters due to physical crosslinking, and allowing the reversible formation and reformation of the network structure to provide self-healing properties via reversible breakage and reformation of ionic bonds [9,10]. Compared to other types of non-covalent bonds, ionic interactions have higher aggregate strength, which facilitates increased tensile strength, toughness, and fracture resistance. But, they also require more external energy to break [11]. Like hydrogen bonding, ionomers effectively behave as an autonomous self-healing material in some applications. An example of this is in a ballistic impact healing application where the damage event provides enough energy in the form of heat generated by friction to complete healing [11,68,69]. Dynamic ionic crosslinking has proven to be an efficient way to heal damages in 3D-printed polymers [39,70]. Liu et al. exploited the dynamic ionic crosslinked network formed between the carboxyl and amino-functional polysiloxanes to ensure the self-healing and reprocessing capabilities of 3D-printed silicone elastomers. This showed excellent healing efficiency of 97% and a healing process that could be repeated multiple times, in addition to repeatedly reprocessing these elastomers and still repairing the damage with over 90% efficiency (Figure 3d) [39].

Polyacrylic acid is often used to achieve autonomous intrinsic self-healing properties in hydrogels that can be used as 3D printing materials, and self-healing hydrogels allow for ion transport while maintaining excellent mechanical stability [21,71,72]. Darabi et al. utilized dynamic ionic interactions between the carboxylic groups of poly(acrylic acid), NH groups of polypyrrole and ferric ions, and a combination of both physical and chemical crosslinking to enable the hydrogel to meet the highest self-healing efficiency while maintaining mechanical stability and electrical conductivity [71].

##### Coordination Interactions

Among various non-covalent interactions, metal–ligand interactions can also form supramolecular networks and have unique properties [7,11]. Coordination complexes are formed between positively charged metal ions and partially negatively charged groups on ligand molecules. There are a large number of accessible metal ions and ligand molecules that make coordination chemistry particularly attractive by careful selection of the combination of ligands and metal ions (Figure 3c) [1,11]. It is possible to tune the bond strength into the desired weak and dynamic, or, with the presence of functional metal ions or ligands and dynamic metal–ligand bonds, polymers can display a variety of advanced functions such as dielectric, magnetic, luminescent, catalytic, and stimulating reactivity [1,7]. For example, by replacing the metal ion with which the pyridine interacts, the Zn–pyridine interaction is strong but not dynamic compared to the Fe–pyridine and Tb–pyridine interactions used for self-healing [10]. The charge on the ligand molecule is usually much smaller than that on the metal ion. Since dipole–ion interactions are weaker than ion interactions, the strength of coordination bonds is weaker even if they can be adjusted within a specific range. These weaker crosslinks allow for the healing of macroscopic damage at mild temperatures without the need for external stimulus [1,11].

Similar to the two non-covalent bonds mentioned above, coordination bonds can also be used to achieve self-healing of 3D-printed materials. Lai et al. used the weak but abundant Zn(II)–carboxylate coordination bond to design polydimethylsiloxane (PDMS) polymers and achieve rigid and healable materials through 3D printing. The coordination equilibrium is temperature sensitive, with the mutual crosslinking of Zn(II) and carboxylate at room temperature. The equilibrium shifts toward the dissociated state when temperature is increased, producing an increasing number of non-cross-linked chains, which can resume crosslinking when the temperature is cooled down again (Figure 3g) [34]. Shi et al. utilized dynamic coordination bonds between bisphosphonate (BP) ligands and calcium ions based on the polysaccharide hyaluronic acid (HA) backbone to design a new hydrogel ink that can be used for extrusion-based 3D printing. Similarly, Wang et al. used ionic interactions between poly(acrylic acid) acid and calcium ions combined with dual-crosslinked networks to develop a multifunctional hydrogel with extraordinary mechanical strength and self-healing efficiency (Figure 3j) [72,73].

### 2.3. Development Status

One strategy for developing more advanced self-healing materials is to combine multiple self-healing mechanisms. This approach is also applicable to developing self-healing materials for 3D printing, as reported in several research papers [10,29,31,33,43,74].

Wu et al. developed a Cu(II)–dimethylglyoxime–urethan-complex-based polyurethane polymer link (Cu–DOU–CPU) with a synergistic triple dynamic bond, including the presence of dynamic covalent bonds (oxime amine bonds) and dynamic non-covalent bonds (metal–ligand bonds and hydrogen bonds), which can enhance both the self-healing properties and the mechanical properties of the material [33]. The relative recovery rate of 3D-printed objects reached 94% in the absence of any external stimuli (Figure 3h) [33].

Xu et al. designed a double-network hydrogel consisting of a chitosan–citrate (CS) network crosslinked by citrate ions via electrostatic interaction and poly(sulfobetaine-co-acrylic acid) (P(SBMA-co-Ac)) network crosslinked by hydrogen bonding between carboxyl groups and ionic interaction between zwitterionic moieties (Figure 3i). This had a self-healing-property of 95.4% [74]. The hydrogel was 3D-printed with good electrical conductivity and sensitivity. It can be used as a strain sensor for detecting human motion, retaining good sensitivity after fracture, and self-healing [74].

Similarly, Chen et al. have produced ionic gels with remarkable self-healing properties by combining dynamic disulfide, hydrogen bonds, and ionic interactions by 3D printing, achieving healing efficiencies of over 95% under heating, and over 99% under UV irradiation, while offering advantages of high elasticity and durability (Figure 3e) [43]. Xu et al. prepared 3D-printed excellent performance composites with self-healing properties that are attributed by a combination of Diels–Alder (DA) reversible covalent bonding and hydrogen bonding [31].

## 3. Additive Manufacturing of Self-Healing Materials

### 3.1. Extrusion-Based AM Processes

Among several 3D printing techniques, extrusion-based AM processes have great potential as multi-material or multifunctional fabrication methods. They are widespread at amateur and professional levels because of their low cost, simple operation, low environmental impact, and relatively high prototyping speed [75,76,77,78,79]. Two of the most common extrusion-based AM processes are FDM and DIW [80].

#### 3.1.1. Fused Deposition Modelling (FDM)

**Figure 4:**
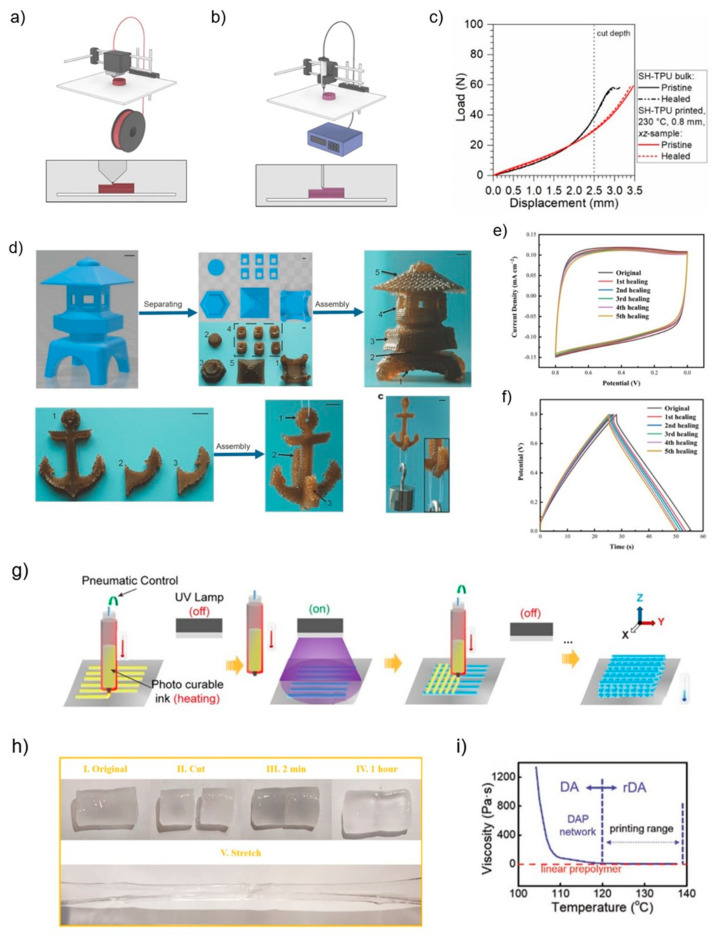
(**a**,**b**) Schematic diagram of fused deposition modelling (FDM) and direct-ink writing (DIW) [80]. (**c**) Compression-cut mechanical characterization of healed FDM-printed and bulk SH-TPU polymers [14]. (**d**) Example of free assembly of 3D-printed parts into complex objects. The numbers in the photographs represent the order of assembly [33]. (**e**) CV curves of the MSC at different damage/self-healing cycles. (**f**) GCD curves of the MSC at different damage/self-healing cycles [21]. (**g**) DIW-based 3D printer equipped with heating elements prints each filament layer followed by shining UV light to cure the resin [35]. (**h**) Digital photographs demonstrating the self-healing process of the hydrogel designed by Wang et al.: I original state; II cut into two pieces; III self-healing in 2 min; IV self-healing in 1 h; V stretching after healing [21]. (**i**) Viscosity of the DAPs and the linear prepolymer as a function of temperature [32].

FDM, also called fused filament fabrication (FFF), is an extrusion-based method in which a continuous filament of polymer melts from a heated nozzle, depositing the molten material in a consecutive, layer-by-layer fashion on a print bed that may also be heated (Figure 4a) [14,81]. The material cools and solidifies upon deposition. Then, the next layer is deposited similarly to build a digitally modelled part iteratively [80].

Polymers used for FDM are typically thermoplastic or nanocomposites, and there are many advantages to adding self-healing properties to the material. FDM 3D-printed polymeric parts have been shown to completely retain their self-healing ability as the bulk self-healing polymer while showing the potential to obtain improved (3D-printed polymeric components) properties such as mechanical properties, durability, damage tolerance, and extensibility [14,30]. For example, Ritzen et al. found through a compression cut test that self-healing thermoplastic polyurethanes (SH-TPU) printed by FDM had the same mechanical and healing behavior as a bulk self-healing polymer, implying that the self-healing property of the polymer was unaffected by processing steps and printing (Figure 4c) [14]. Peng et al. reported that the mechanical properties and extensibility of the material were considerably enhanced when the morphology was preferentially oriented along the printing direction [30].

Nevertheless, the FDM method results in weak interlayer adhesion during layer-by-layer deposition, possibly due to the high viscosity of the solvent-free resin. This disadvantage may tend to cause cracks and deformations during use, thus, significantly reducing the service life of the manufactured object. This problem can be solved using functional materials with a higher affinity for fusion and layer adhesion, such as self-healing materials [29,32,33]. Zhou et al. used DA-based polymers (DAPs) that dissociate into low-viscosity liquids when their temperature exceeds that of its dissociation temperature (T_D_), successfully addressing current significant challenges in FDM (Figure 4i) [32]. Similarly, in another report, a polymer ink with synergetic multiple dynamic bonds, including reversible coordination and hydrogen bonds used by Zuo et al., was also developed to optimize FDM printing conditions [33]. It reported that the method increases interlayer adhesion and reduces the number of support structures available by taking advantage of room-temperature self-healing property to assemble 3D-printed blocks into large complex objects, with the added benefit of decreasing the complexity and cost (Figure 4d) [33]. O’Harra et al. introduced ionic interactions and H-bonding into elastomeric materials printed by FDM, which provided the opportunity to not only eliminate the inherent weaknesses of FDM 3D printing but also to retain the homogeneity and desirable thermophysical properties of the material [29].

#### 3.1.2. Direct-Ink Writing (DIW)

DIW, also called liquid deposition modelling (LDM), utilizes a pneumatic, piston, or screw to extrude the print material through a nozzle or syringe needle (Figure 4b) [36,82]. Some curing processes can be performed by thermal and photopolymerization curing or dispensing two reactive components using mixing nozzles to facilitate the deposition and subsequent stabilization of the material (Figure 4g) [80,83,84].

Like FDM, the nozzle or syringe needle can be moved over the build surface in three dimensions at a constant height. The extruded materials are joined together layer-by-layer to form the final 3D construct [80,84]. DIW printing inks require unique rheological properties, such as low viscosity and shear-thinning, to ensure continuity of printing without resorting to excessively high pneumatic or mechanical pressure, and to stop the flow or restore mechanical integrity after extrusion [80,83]. Compared to thermoplastic printing in FDM, DIW may be utilized to print thermoset materials that are inherently less processable. Introducing self-healing properties to thermoset materials printed via DIW allows for the retention of mechanical strength and durability of traditional thermosets, and endows the materials with additional self-healing properties and processability [16,36]. In Yuan’s report, thermally reversible Diels–Alder (DA) was used in a thermoset to develop a self-healing ink for DIW. The self-healing properties in materials enable high strength recovery of up to 85%, and repeatable healing without a significant decrease in healing performance. The material can also be reprocessed and remolded. In addition, the relatively low viscosity of the ink eliminates voids between the printed filaments, resulting in high tensile strength [36]. Despite the many advantages of DIW for manufacturing thermoset materials, challenges remain in developing printing inks with rheological optimization [36].

Extant research has shown that results have been achieved with DIW-printed self-healing materials. Wang et al. developed a self-healing and highly stretchable hydrogel achieved by dynamic borate ester and multi-network hydrogen bonds (Figure 4h). After direct DIW printing, the micro-supercapacitor (MSC) showed initial structural self-healing properties, and enabled rapid electrochemical restoration with little change in cyclic voltammetry (CV) curves and galvanostatic charge/discharge (GCD) curves after multiple physical damage/healing cycles. This is beneficial for the study of self-healing hydrogel systems in portable wearable electronic devices (Figure 4e,f) [21].

Since Kuang et al. first combined the attributes of shape memory and self-healing with 3D printing in 2018, shape-memory-assisted self-healing has received increasing attention [35]. This makes sense, as the introduction of self-healing improves the damage accumulated during the repeated deformation–recovery process of the printed part without external healing agents [16,85]. In Kuang’s report, the embedded semicrystalline thermoplastic plays a dual role as a switching phase for shape memory and a healing agent for self-healing behavior, and complex structures with functional properties such as shape memory and self-healing were printed by DIW [35]. Similar to Kuang’s experiment, Zhang et al. introduced the DA reaction into SMPs, and obtained final printed objects with designed shapes, higher quality, and precise self-healing properties by DIW [16].

### 3.2. Vat Photopolymerization

Vat photopolymerization, such as SLA and DLP 3D printing, avoid the problems of extrusion printing with extensive rheological optimization of inks, and the possibility of displaying warped and slightly distorted planes. They also have better print resolution and higher efficiency [25,37,44].

**Figure 5:**
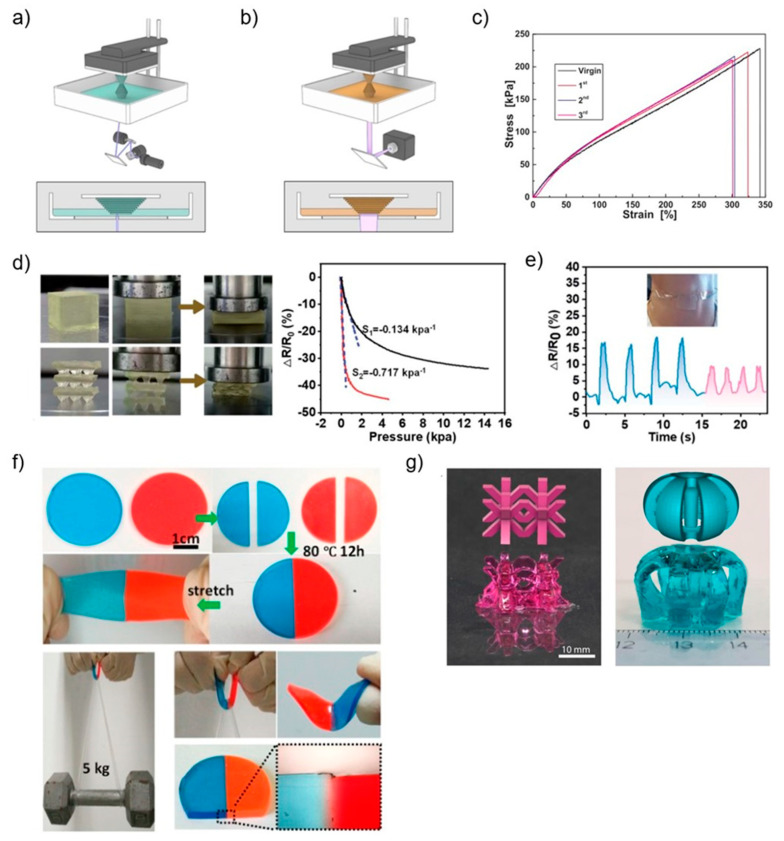
(**a**,**b**) Schematic diagram laser stereolithography (SLA) and digital light processing (DLP) [80]. (**c**) Liu et al. designed SLA-printed silicone elastomers with 97% healing efficiency [39]. (**d**) Comparison of the pressure sensitivity of the planar sensors (S_1_) and micro-structured (S_2_). Pressure sensitivity (S) is expressed as the relative resistance change ratio over pressure (ΔR/R_0_)/P [43]. (**e**) The micro-structured pressure sensor senses the subtle muscle movements of the throat during the swallowing of water (the first four cycles) and saliva (the last four cycles) [43]. (**f**) Image of the polyurethane elastomer designed by Li et al. cut into two pieces, connected, healed for 12 h at 80 °C and, finally, subjected to first stretching manually to a large deformation and then to a 5 kg weight lifting test; optical microscope image of the healing interface of the healed elastomer [41]. (**g**) Complex structures with flat surfaces and sharp edges printed by DLP. Body-centered cubic lattice-like structure printed with methyl red sodium salt dye (**left**); axisymmetric structure with central pillar printed with brilliant green dye (**right**).

Vat photopolymerization is a continuous, layered technique for fabricating a part by using UV or visible light to project a 2D pattern onto a liquid photopolymer resin, with the first layer of the part being cured directly onto the build surface and each subsequent layer being cured onto the previous layer, repeating the process until the manufacturing is complete. SLA and DLP are subdivided by light source configuration—in SLA 3D printing, the laser scans point-by-point and cures the photopolymer resin to complete each layer of the pattern; while in DLP systems, UV light can cure the entire patterned area of each layer in a single exposure (Figure 5a,b) [44,80].

Resins for vat photopolymerization usually consist of monomers, oligomers, functionalized polymers, and photoinitiators, and the resulting resin must be translucent to the light source used for curing to allow UV light to penetrate and cure the designed layer height. At the same time, the viscosity must be manageable to ensure fluidity [80].

Three-dimensional photopolymerization is a free radical polymerization reaction that occurs in the presence of a photoinitiator using light as the stimulus, and (meth)acrylates and (meth)acrylamides are usually used because of their fast reaction rate, which allows for faster conversion of reactive liquid resins into solid materials [86].

#### 3.2.1. Stereolithography (SLA)

Photopolymerization is widely used because of some excellent characteristics. For example, the leading additive manufacturing methods for silicone elastomers are extrusion 3D printing and photopolymerization. These are popular because they break some limitations of extrusion 3D printing, and also have high efficiency, higher resolution, and better surface quality [39,87]. Liu et al. have fabricated self-healable and reprocessed silicone elastomers by SLA 3D printing and achieved a healing efficiency of 97% (Figure 5c) [39]. The first 3D-printed structure with extrinsic self-healing properties was also achieved by SLA, where Sanders et al. successfully printed 3D self-healing composites by adding self-healing capsules to SLA resin to provide a solvent-welding effect [37].

Additionally, 3D printing techniques based on vat photopolymerization have been widely used to produce high-resolution thermoset materials. Objects created with vat photopolymerization techniques offer superior thermal stability and quality due to the formation of covalent crosslinks between the layers [38]. Zhang et al. successfully printed thermoset materials with self-healing properties, excellent mechanical properties, and high modulus by incorporating a reversible addition–fragmentation chain transfer agent into the SLA resin [86]. Similarly, Durand-Silva et al. successfully used SLA to print thermoset polymers with self-healing properties enabled by Diels–Alder reactions [38].

#### 3.2.2. Digital Light Processing (DLP)

Because DLP can irradiate and cure one layer at a time, it has a shorter print time than SLA, and a more comprehensive range of applications. For example, in 2022, Zhang et al. took advantage of the high printing accuracy of DLP 3D printing technology and applied it to the fabrication of micro-structured pressure sensors that meet the requirements of ultrahigh elasticity and durability and remarkable self-healing properties, while significantly improving the sensitivity of the sensors to detect complex muscle movements and subtle motions (Figure 5d,e) [43]. In addition, the printable ionic gel possesses tunable mechanical properties due to the easy modification of the ratio of the individual components of the DLP resin [43].

For the first time in 2019, Li et al. fabricated a self-healing polyurethane elastomer by DLP 3D printing, which can heal up to 95% efficiency and multiple times because of hydrogen and disulfide bond metathesis. The polyurethane elastomer has a good prospect for application due to ease of manufacture, excellent performance, high precision, and complex structure (Figure 5f) [41]. Additionally, there are also improvements in the additive manufacturing of hydrogels, which were previously only processed by extrusion-based additive manufacturing techniques with limited freedom in terms of design and resolution.

However with DLP 3D printing, it is possible to print bespoke structures with overhanging, hollow features, and high precision without the need for support materials [44]. For example, Caprioli et al. in 2021 used the dispersive forces between materials to effectively interact to overcome the inherent incompatibility between vat photopolymerization and self-healing properties, printing complex structures with sharp edges and a self-healing efficiency of 72% via DLP (Figure 5g) [44].

## 4. Applications and Perspectives

In recent years, the field of additive manufacturing has witnessed remarkable achievements in the printing of self-repairing polymers and composites. These advancements have paved the way for groundbreaking applications spanning a wide range of industries. Notably, aerospace engineering has been revolutionized by the emergence of 3D-printed polymer matrices capable of autonomously mending microcracks and damages found in structural components [88,89]. This development has significantly impacted maintenance strategies while ensuring enhanced longevity and safety for critical aircraft parts. Similarly, the biomedical industry has experienced a paradigm shift with the fabrication of customized biocompatible implants possessing inherent self-healing properties [90,91]. The introduction of these innovative medical devices minimizes invasive replacement procedures while maximizing patient comfort levels. By harnessing this technology, patients can benefit from prolonged use without compromising their quality of life. The automotive industry is yet another beneficiary where significant advantages have emerged through the utilization of self-repairing polymer composites for impact-resistant car parts, enabling vehicles to be more resilient to external forces encountered on roads or during accidents [92]. These developments highlight not only the immense potential but also underscore key benefits such as increased sustainability, cost-effectiveness, and resilience across diverse sectors when combining additive manufacturing techniques with self-healing polymers and composites [93].

Although breakthroughs have been made in the field over recent years, its potential is hindered by several limitations. For example, the selection of appropriate self-healing mechanisms, which involves careful consideration of trade-offs. Chemical mechanisms exhibit commendable healing efficiency but may necessitate specific activation conditions that can potentially impact AM processes adversely. On the other hand, physical mechanisms like shape-memory polymers offer greater compatibility with AM techniques. The choice between microcapsules and vascular networks depends on the unique demands posed by each application. Microcapsules enable localized healing capabilities while vascular networks provide broader avenues for restoration. Material development poses significant challenges due to the need for harmonizing printability with mechanical performance, thereby demanding innovative formulations. Achieving material compatibility between the healing agent and polymer matrix emerges as a critical factor in this pursuit; furthermore, scaling up 3D printing for large-scale applications remains an obstacle that needs to be overcome. While there are many advantages to manufacturing self-healing polymers by AM, the inherent material properties present unique processing challenges for the field. For extrusion-based AM processes, the printing ink has to meet the rheological requirements while ensuring that the printed objects are sufficiently rigid. In addition, 3D-printed self-healing polymers with a high crosslinking density can reduce the molecular motion of the polymer chains, which can affect the self-healing effect. These require both polymer chemistry innovations and printer design to achieve a balance between performance. Until this can be achieved, the benefits of manufacturing self-healing polymers by AM cannot be fully taken advantage of and will remain theoretical.

The integration of multiple self-healing mechanisms with hybrid 3D printing represents another highly promising avenue for further advancing the utilization of self-healing polymers and composites. By introducing redundancy in the healing process, this approach not only enhances healing efficiency but also ensures greater reliability in critical applications. Additionally, it enables the tailored integration of diverse mechanisms, allowing for optimized material performance based on specific conditions and requirements. The versatility offered by hybrid 3D printing allows for the precise incorporation of these mechanisms into intricate structures, pushing the boundaries of what can be achieved with self-healing materials. This advancement has profound implications across a myriad of industries that demand durable and resilient materials. Notably, it offers sustainability benefits through waste reduction, on-demand production capabilities, and extended product lifecycles. While challenges such as material compatibility and design complexities must be addressed, the synergistic combination of multiple self-healing mechanisms with hybrid 3D printing holds potential to revolutionize material design and application.

This paper summarizes the status quo of developments in self-healing mechanisms for self-healing polymers, as well as its advantages and its disadvantages. It also highlights examples of applications of the main 3D printing methods used to manufacture polymers. However, research into the field is far from maturity—damaged parts are repaired but not fully restored to their original surface, and damage cure has only been demonstrated at the laboratory level. While there appears to be great potential for real-world applications of self-healing polymers, it will be some time before its projected economic and environmental impact materializes [94,95]. Functional self-healing polymers are a growing trend—combining self-healing materials with additional functions such as shape memory property [96,97,98,99,100,101,102], electrical conductivity [103,104,105], etc., could further expand the market for applications.

## Figures and Tables

**Table 1:** Brief summary of the existing literature on printable self-healing materials.

	Extrinsic Healing			Intrinsic Healing				
	Microcapsule	Microvascular	Nanoparticles	Diels–Alder Reaction	Disulfide	Hydrogen Bonds	Ionic Interactions	Coordination
**Fused deposition modeling (FDM)**		O’Harra et al. (2020) [29]	Peng et al. (2021) [30]	Bi et al. (2022) [31] Zhou et al. (2020) [32]		O’Harra et al. (2020) [29] Bi et al. (2022) [31] Zuo et al. (2021) [33]	O’Harra et al. (2020) [29]	Zuo et al. (2021) [33] Lai et al. (2018) [34]
**Direct-ink writing (DIW)**			Kuang et al. (2018) [35]	Zhang et al. (2019) [16] Yuan et al. (2020) [36]		Wang et al. (2021) [21]	Wang et al. (2021) [21]	
**Stereolithography (SLA)**	Sanders et al. (2019) [37]			Durand-Silva et al. (2021) [38]			Liu et al. (2020) [39]	
**Digital light processing (DLP)**		Kuang et al. (2018) [35]	Zhang et al. (2019) [40]		Li et al. (2019) [41] Miao et al. (2022) [42] Zhang et al. (2022) [43]	Zhang et al. (2022) [43] Caprioli et al. (2021) [44]	Zhang et al. (2022) [43]	
